# Epigenetic Regulatory Effect of Exercise on Glutathione Peroxidase 1 Expression in the Skeletal Muscle of Severely Dyslipidemic Mice

**DOI:** 10.1371/journal.pone.0151526

**Published:** 2016-03-24

**Authors:** Albert Nguyen, Natacha Duquette, Maya Mamarbachi, Eric Thorin

**Affiliations:** 1 Department of Pharmacology, Faculty of Medicine, Université de Montréal, Montreal, Quebec, Canada; 2 Montreal Heart Institute, Research Center, Montreal, Quebec, Canada; 3 Department of Surgery, Faculty of Medicine, Université de Montréal, Montreal, Quebec, Canada; University of Debrecen, HUNGARY

## Abstract

Exercise is an effective approach for primary and secondary prevention of cardiovascular diseases (CVD) and loss of muscular mass and function. Its benefits are widely documented but incompletely characterized. It has been reported that exercise can induce changes in the expression of antioxidant enzymes including Sod2, Trx1, Prdx3 and Gpx1 and limits the rise in oxidative stress commonly associated with CVD. These enzymes can be subjected to epigenetic regulation, such as DNA methylation, in response to environmental cues. The aim of our study was to determine whether in the early stages of atherogenesis, in young severely dyslipidemic mice lacking LDL receptors and overexpressing human ApoB100 (LDLR^-/-^; hApoB^+/+^), exercise regulates differentially the expression of antioxidant enzymes by DNA methylation in the skeletal muscles that consume high levels of oxygen and thus generate high levels of reactive oxygen species. Expression of *Sod2*, *Txr1*, *Prdx3* and *Gpx1* was altered by 3 months of exercise and/or severe dyslipidemia in 6-mo dyslipidemic mice. Of these genes, only *Gpx1* exhibited changes in DNA methylation associated with dyslipidemia and exercise: we observed both increased DNA methylation with dyslipidemia and a transient decrease in DNA methylation with exercise. These epigenetic alterations are found in the second exon of the *Gpx1* gene and occur alongside with inverse changes in mRNA expression. Inhibition of expression by methylation of this specific locus was confirmed *in vitro*. In conclusion, *Gpx1* expression in the mouse skeletal muscle can be altered by both exercise and dyslipidemia through changes in DNA methylation, leading to a fine regulation of free radical metabolism.

## Introduction

Chronic inflammatory diseases such as atherosclerosis are characterized in part by high levels of free radicals, inducing oxidative stress [1]. This oxidative stress targets DNA, lipids and proteins, altering cellular functions and leading to organ failure [2]. To maintain a healthy *redox* equilibrium, cells normally express endogenous antioxidant enzymes including peroxiredoxins (Prx), thioredoxins (Trx), superoxide dismutases (SOD) and gluthatione peroxidases (Gpx). These enzymes inactivate reactive oxygen species (ROS) and maintain them to physiological levels. ROS are indeed signalling molecules and natural by-products of the metabolic machinery and are therefore necessary for cellular function [3].

ROS are poorly regulated in the presence of a chronic inflammatory state such as diabetes, cancer and dyslipidemia [4]. With inflammation, the *redox* equilibrium tips towards the generation of free radicals by simultaneous occurrences of pro-oxidative events, including dysregulation of ROS-generating enzymes such as NADPH oxidases (NOX) normally involved in cell survival, growth and death [5], as well as regulators of the mitochondrial respiratory chain such as uncoupling proteins (UCP) [6]. The primary consequence of a rise in oxidative stress is a vascular endothelial dysfunction, a marker of future cardiovascular events [7]. However, clinical trials using antioxidants in patients with cardiovascular diseases (CVD) have provided controversial and still inconclusive results [8, 9]. In contrast, while physical exercise paradoxically increases ROS production both in animal studies [10] and humans [11], physical training is an excellent primary and secondary prevention strategy in CVD [12] and delay the loss muscle mass and function (sarcopenia) [13], including in patients suffering from atherosclerosis [14, 15]. While an acute bout of exercise increases ROS production, chronic regular exercise upregulates antioxidant defenses [16–18].

Little is known, however, about the molecular mechanisms increasing stress resistance. During physical exercise, the main source of ROS is the skeletal muscle [13]. Muscles of the lower limbs are high producers of mitochondria-derived metabolites, and it is therefore likely that ROS arising from the metabolic activity of the skeletal muscle could potentially damage the vascular endothelium if the appropriate defense mechanisms are not maintained properly [13]. This supposes that genes coding for antioxidant proteins are partly regulated by the level of physical activity.

Epigenetic regulation of gene expression in response to environmental stimuli is well documented [19]. In mammals, the addition of a methyl group to a cytosine preceding a guanine (CpG), usually leads to gene silencing if located in CpG-dense promoter regions. However, the regulatory effects of methylation in the gene body are more ambiguous [20–22]. DNA methylation is a dynamic process that can respond to most extracellular cues including diet [23] and physical activity [24]. This sensitivity of DNA methylation to environmental signals is suggested to contribute to the development of various pathological conditions since gene-specific and genome-wide DNA methylation have been linked to a wide variety of disease states ranging from inflammatory [25] and CVD [26] including atherosclerosis [27, 28] and skeletal muscle [29]. This highlights the importance of epigenetic plasticity to the environment.

Studies have shown that antioxidant enzymes could be induced or repressed by methylation of regions in the corresponding gene, while aberrant methylation patterns have been associated with pro-oxidative and pro-inflammatory diseases [30–32]. We therefore hypothesised that in young mice, severe dyslipidemia would alter antioxidant gene expression while chronic physical exercise would maintain it, in part due to epigenetic regulation. In this study, we demonstrate the dynamic nature of antioxidant genes expression in the mouse skeletal muscle in sedentary and active dyslipidemic mice. Our results show for the first time that *Gpx1* expression is associated with changes in DNA methylation in a specific region in the gene body. Hence, physical exercise influences *Gpx1* gene expression through epigenetic regulation and may ultimately contribute to the cellular defense against metabolic stress.

## Materials and Methods

**Fig 1:**
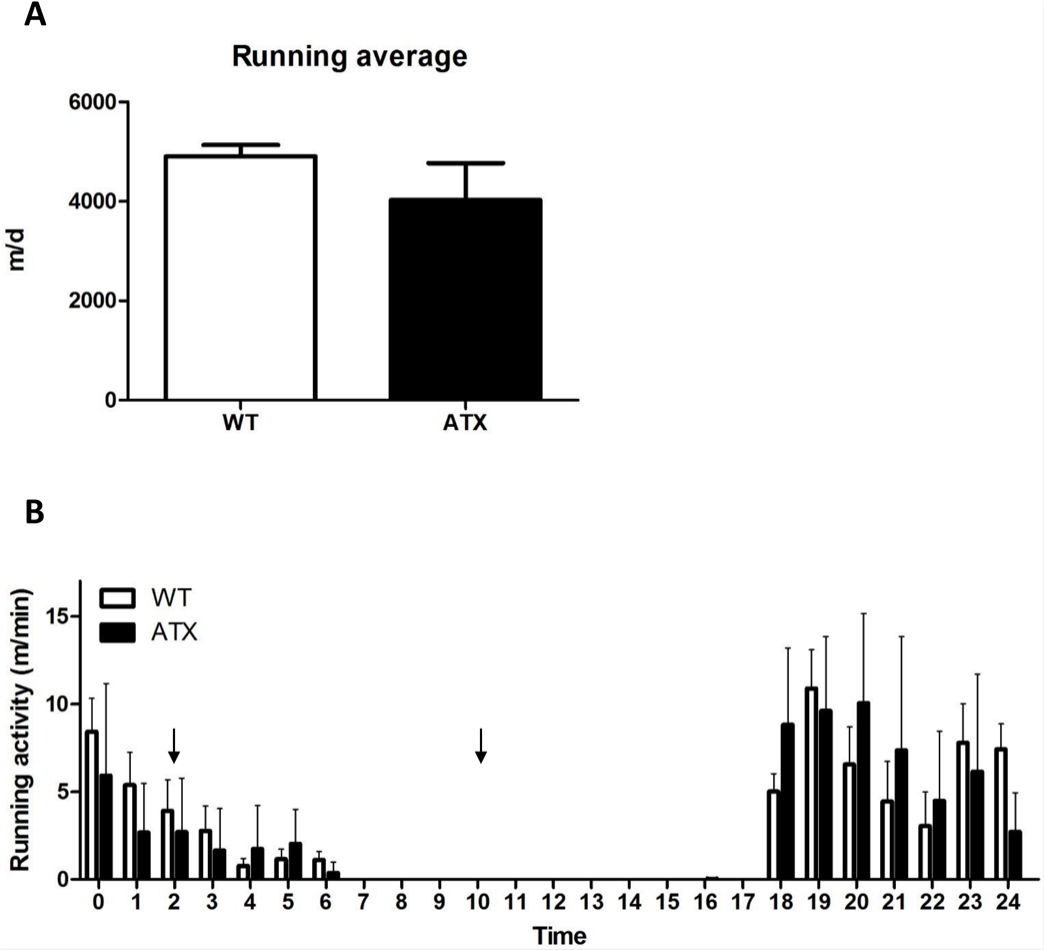
Voluntary running activity is similar between WT and ATX. (A) Average of the daily running distance over the course of the 3 months exposure to voluntary exercise for both WT and ATX groups. (B) Typical distribution of the running activity during an arbitrary day of voluntary exercise. Arrows indicate the two different times of sacrifice (2:00 AM and 10:00 AM).

Approval by the Montreal Heart Institute Animal Ethical Committee (#R2014-62-02) was given for all animal experiments, which were performed in accordance with the *Guide to Care and Use of Experimental Animals* (vol.1, 2^nd^ ed., 1993) of the Canadian Council on Animal Care. No animals became ill or died prior to the experimental endpoint. Experiments were conducted on the femoral artery and the skeletal muscle (soleus and gastrocnemius) isolated from 6-months old (6-mo) male C57/bl6 control wild type (WT) compared to severely dyslipidemic and spontaneously atherosclerotic (ATX) mice. Transgenic LDLr^-/-^:hApoB^+/+^ ATX mice display high levels of cholesterol, they spontaneously develop atherosclerotic lesions (under a normal diet) and endothelial dysfunction in the aorta, carotids and renal arteries [33, 34]. Both 3-mo WT and ATX mice were randomly assigned to two groups; one remained in control sedentary (SED) conditions (n = 10) and one was exposed to 3 months of voluntary exercise (EX) (n = 10). To this end, mice were kept individually in cages instrumented with a running wheel (Lafayette Instrument Company, Lafayette, IN) [34]. Heart rate, systolic and diastolic blood pressure were monitored weekly by tail-cuff (Kent Scientific Corporation, Torrington, CT). Mice were studied at 6-mo and were sacrificed after anesthesia with a 1:1 mixture of Xylazine (Bayer Inc, Toronto, ON, Canada) and Ketamine hydrochloride (Bioniche, Belleville, ON, Canada) at two different times of the day, either at 10:00 AM while being inactive or at 2:00 AM, i.e. during their running time (Fig 1).

### DNA and RNA extraction

Upon sacrifice, skeletal muscle tissues were harvested and snap-frozen. Total DNA and RNA were extracted using Qiagen RNeasy mini kit and DNeasy Blood & tissue kit (Qiagen, Toronto, ON), respectively, following manufacturer’s protocol.

### Gene expression

Total RNA was reverse transcribed with RT-MMLV (Invitrogen, Burlington, ON) and amplified using EvaGreen (Applied Biological Materials, Richmond, BC) for quantitative real-time PCR. Target genes *UCP2/3*, *NOX2/4*, *Trx1*, *Sod2*, *Prdx3*, *Gpx1* and the internal control, cycloA, were amplified with gene specific primers (S1 Table).

### DNA methylation quantification

Following extraction, DNA was converted by bisulfite reaction using the EZ DNA Methylation-Gold kit (Zymo Research, Irvine, CA). Global DNA methylation was measured by ELISA with the 5-mC DNA ELISA kit (Zymo Research). Gene-specific DNA methylation was quantified by EpiTYPER assay (Sequenom, San Diego, CA), as previously described [35]. We chose to target regions identified as CpG islands, by the UCSC Genome Browser at http://genome.ucsc.edu/ [36], found in our genes of interest with bisulfite-specific primers (S2 Table) required for the assay.

### Cloning of pCpG free-Gpx1 vector

To study the regulatory consequence of *in vitro* methylation of the targeted CpG island, we first used the commercially available reporter plasmid pCpGfree-promoter as the backbone (Invivogen, San Diego, CA). This plasmid containing both a sequence for secreted luciferase and a promoter devoid of CpG dinucleotides, which renders it insensitive to DNA methylation. We then synthetized the Gpx1 CpG island region by PCR amplification using forward 5’-TATAAGCTTGAAGTGAATGGTGAGAAGGCTCACCCGC-3’ and reverse 5’- ATATGTACAACCAAGCCAATGCCAGGGCCGCCTTAG-3’ primers on mouse genomic DNA as a template. This newly generated CpG-rich sequence was then inserted in the pCpG free-promoter plasmid using HindIII and BsrGI restriction sites. We named the resulting vector “pCpG free-Gpx1” *(see “*results*”)*.

### *In vitro* methylation, transient transfection and Luciferase assay

Cloned vectors were isolated by Qiagen QIAprep Spin Miniprep kit (Qiagen). M. SssI CpG methyltransferase (New England Biolabs, Frankfurt, Germany) was used for *in vitro* methylation according to manufacturer’s instructions. Methylated DNA was then purified using the QIAquick gel extraction kit (Qiagen) and quantified by NanoDrop (Thermo Scientific NanoDrop products, Wilmington, DE). Methylation was confirmed by digestion with the methylation-sensitive restriction enzymes *Hha*I and *Hpa*II. HEK293 cells grown to confluence on 96-well plates were transfected with the pCpG free-Gpx1 vector using Lipofectamine 2000 (Invitrogen). 24 h after transfection, luciferase activity was measured with the QUANTI-Luc reagent (Invivogen, San Diego, CA) by luminescence detection. Promoter activity was normalized to the total amount of protein measured by a Bradford assay (Biorad, Hercules, CA).

### Oxidative stress quantification

The fluorescent probe dihydroethidium (DHE) (Sigma-Aldrich Canada, Oakville, ON, Canada) was used to indirectly assess the amount of superoxide production. DHE is a dye that will bind to DNA once it is oxidized by superoxide. An antibody against 4-hydroxynonenal (4-HNE) (Abcam Inc, Toronto, ON, Canada) was used to detect 4-HNE expression, a marker of lipid peroxidation, by immunostainig. Tissue cross-sections were obtained from OCT-preserved skeletal muscle and confocal images were taken.

### Statistical Analysis

Results are presented as mean±SEM of (n) mice. Two-way ANOVA (with Bonferonni post-tests) and unpaired t-tests were used where applicable to test the differences between conditions. A p value of p<0.05 was considered statistically significant.

## Results

### Phenotypic effects of exercise on severely dyslipidemic mice

We previously reported that LDLr^-/-^; hApoB^+/+^ atherosclerotic (ATX) mice exhibit severe dyslipidemia: compared to wild-type (WT) mice, in 6 months old ATX mice plasma levels of total cholesterol, LDL-cholesterol and triglycerides are ~7, ~13 and ~10 folds higher, respectively [34, 37, 38]. In addition, 3 months of voluntary running had no influence on the observed severe dyslipidemia in ATX mice [34]. At 6-mo, as expected resting systolic and diastolic blood pressures were higher in ATX mice when compared to WT, and this was not affected by voluntary exercise (Table 1). The heart rate was similar across all groups (Table 1). Severe dyslipidemia did not interfere with the running capacity of ATX mice; they ran an average of 4.3 ± 0.4 km/d over the course of the study compared to 4.7± 1.0 km/d for WT mice (p>0.05) (Fig 1A). Both WT and ATX mice ran during the night (Fig 1B).

**Table 1:** Hemodynamic parameters in wild type and dyslipidemic mice exposed or not to a 3 months period of voluntary exercise.

	n	WT SED	WT EX	ATX SED	ATX EX
Systolic	10	115 ± 7	129 ± 5	156 ± 5 *	160 ± 6 *
(mmHg)					
Diastolic	10	84 ± 8	95 ± 5	121 ± 5 *	126 ± 5 *
(mmHg)					
HR	10	605 ± 21	595 ± 18	651 ± 20	640 ± 14
(beats/min)					

Systolic and diastolic blood pressure (mmHg) and heart rate (HR; beats/min) were measured by tail-cuff. Date are mean ± SEM of 10 mice. WT: wild type; ATX: dyslipidemic mice; SED: sedentary; EX: exercise

*: p<0.05 vs. WT (Two-way ANOVA).

### Dyslipidemia and exercise influence the expression of ROS generating enzymes in the skeletal muscle

**Fig 2:**
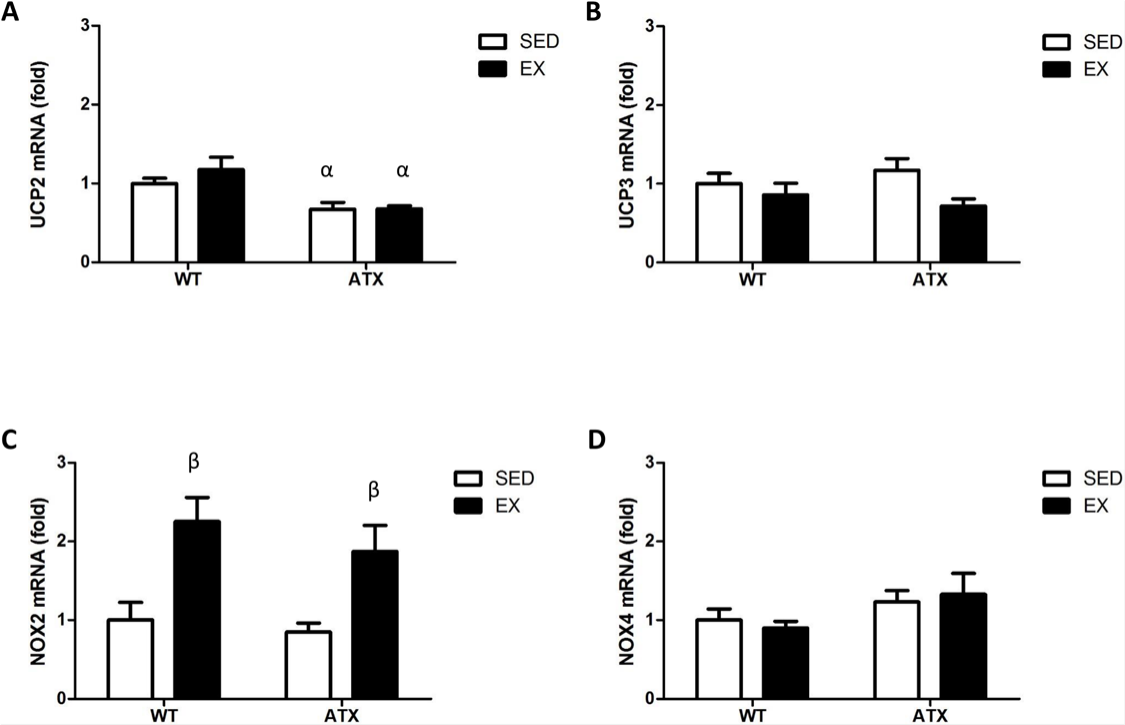
Dyslipidemia and exercise-induced changes in ROS-generating enzymes expression. mRNA levels of *UCP2*, *UCP3*, *NOX2* and *NOX4* in the skeletal muscle of wild type (WT) and dyslipidemic (ATX) mice in the sedentary (SED) and exercise (EX) groups. UCP2/3: uncoupling protein (mitochondrial proton carrier) 2/3; NOX2/4: NADPH oxidase 2/3. Data are mean ± SEM, n = 9–10 mice. α: p<0.05 *vs*. WT; β: p<0.05 *vs*. SED (Two-way ANOVA).

To evaluate the level of expression of endogenous producers of ROS, we quantified the expression of *NOX* and mitochondrial *UCP*. *UCP2* mRNA levels was significantly decreased in ATX mice when compared to WT for both sedentary and EX groups (Fig 2A). *UCP3* expression did not vary between groups (Fig 2B). We did not find differences in expression between WT and ATX sedentary mice for both *NOX2* and *NOX4*. However, *NOX2* expression was significantly stimulated by exercise in both WT and ATX groups (Fig 2C), while *NOX4* expression did not change with exercise (Fig 2D).

### Dyslipidemia and exercise induce changes in antioxidant enzymes expression in the skeletal muscle

**Fig 3:**
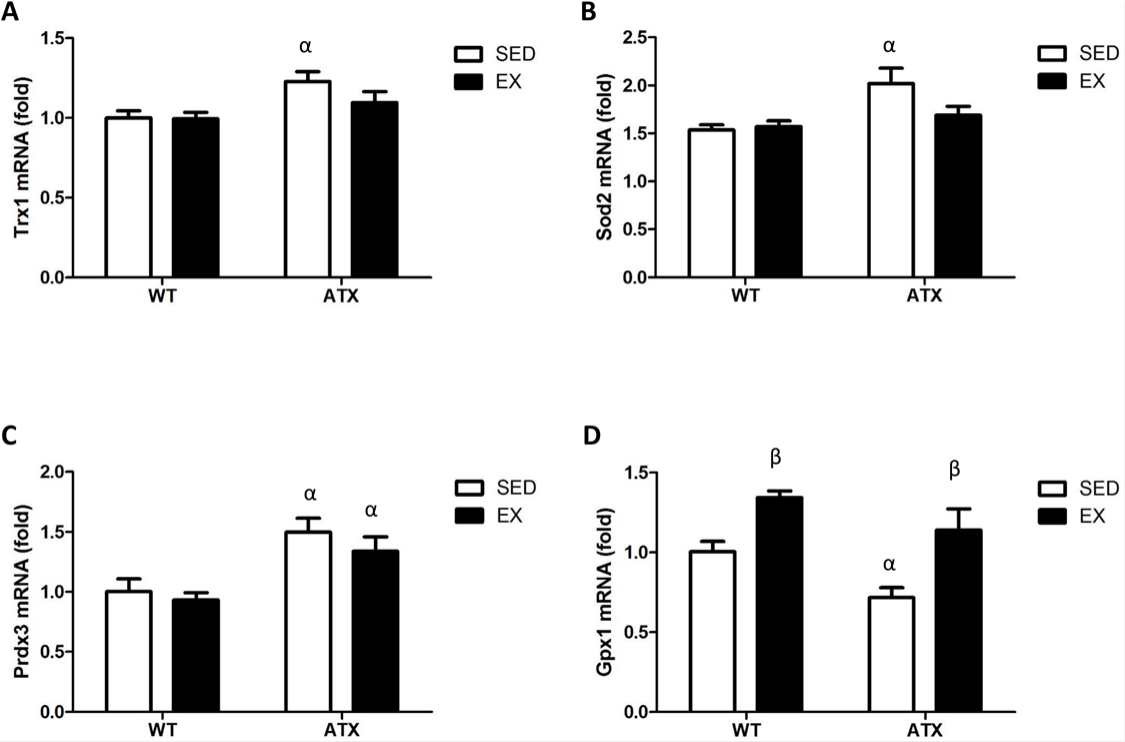
Dyslipidemia and exercise-induced changes in antioxidant enzymes expression. mRNA levels of *Trx1*, *Sod2*, *Prdx3* and *Gpx1* in the skeletal muscle of wild type (WT) and dyslipidemic (ATX) mice from the sedentary (SED) and exercise (EX) groups. Trx1: thioredoxin 1; Gpx1: glutathione peroxidase 1; Prdx3: peroxiredoxin 3; Sod2: superoxide dismutase 2, mitochondrial. Data are mean ± SEM, n = 6–10 mice. α: p<0.05 *vs*. WT; β: p<0.05 *vs*. SED (Two-way ANOVA).

We next examined some of the antioxidant enzymes expressed in the skeletal muscle. In sedentary conditions, both *Trx1* (Fig 3A) and *Sod2* (Fig 3B) were up-regulated in ATX when compared to WT. This change in expression associated with dyslipidemia was prevented by exercise (Fig 3A and 3B). *Prdx3* was up-regulated in both sedentary and exercising ATX mice when compared to the corresponding WT group (Fig 3C). *Gpx1* mRNA expression was significantly lower in sedentary ATX mice in comparison to sedentary WT mice (Fig 3D). *Gpx1* mRNA expression was also higher in exercising WT and ATX mice when compared to sedentary WT and ATX mice (Fig 3D). These changes in Gpx1 mRNA expression are also reproduced in Gpx1 protein expression of WT, but not ATX mice (S1 Fig). Therefore, in ATX mice at this young age corresponding to the early stages of atherosclerosis, skeletal muscle antioxidant enzymes are up-regulated except for *Gpx1*.

### Differential DNA methylation of *Gpx1* in the skeletal muscle of ATX mice

**Fig 4:**
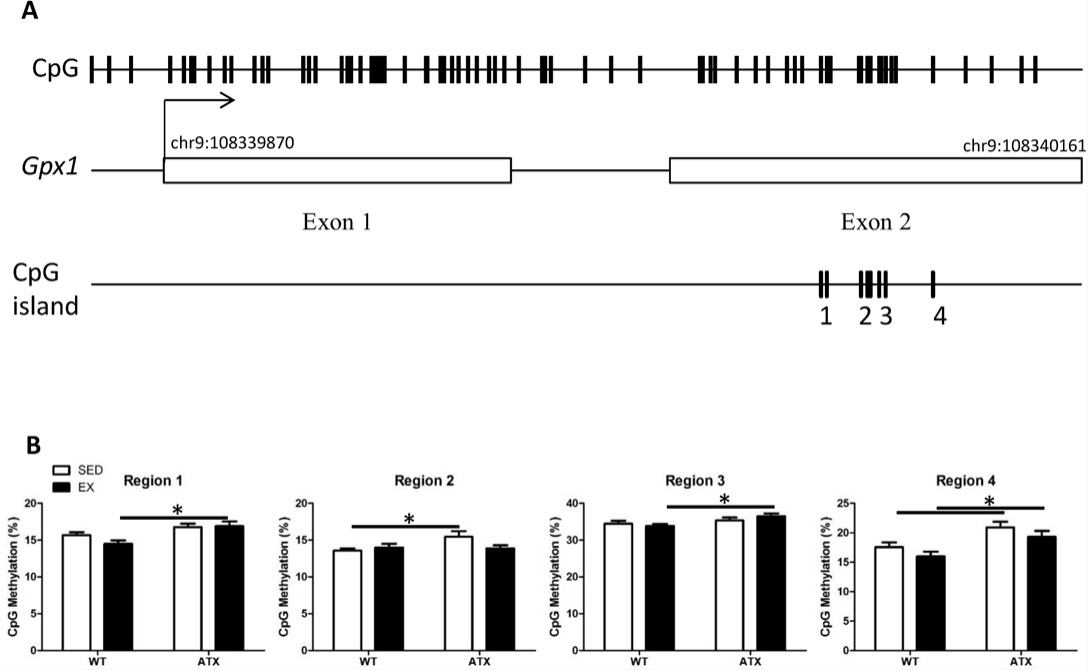
Dyslipidemia induces methylation changes in the *Gpx1* gene body. (A) Glutathione peroxidase 1 gene targeted for methylation quantification; representation of (top track) all CpG sites located in *Gpx1* gene, (middle track) the coordinates of exons and (bottom track) the CpG islands containing the four regions covered by DNA methylation quantification. (B) Methylation percentage of four regions covering CpGs in the skeletal muscle of wild type (WT) or dyslipidemic (ATX) mice under sedentary (SED) or exercise condition (EX). Data are mean ± SEM, n = 7–8 mice. *: p<0.05 (Two-way ANOVA).

We targeted CpG islands located in genes coding for the aforementioned antioxidant enzymes in order to test the hypothesis that epigenetic regulation contributes to the changes in gene expression. DNA methylation was below detection levels for *Trx1* and *Prdx3*, while *Sod2* methylation (>10%) did not vary between conditions (S2 Fig). Quantification of *Gpx1* gene methylation by regions regrouping a single or more CpGs revealed differential methylation levels in the second exon (Fig 4A). Levels of methylation were significantly higher in ATX mice when compared to WT mice for both sedentary (regions 2 and 4) and EX conditions (regions 1, 3 and 4) (Fig 4B). Three months of voluntary exercise did not, however, alter the methylation of this gene, neither in WT nor in ATX mice (Fig 4B).

### Transient changes in DNA methylation following exercise

**Fig 5:**
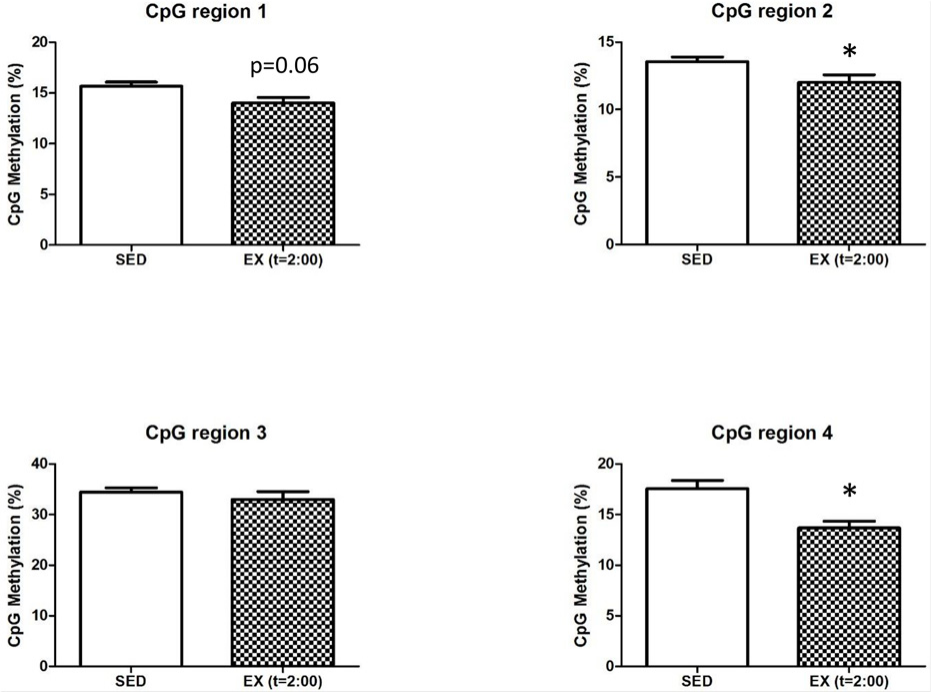
Exercise induces a temporary decrease in DNA methylation. Methylation percentage of the four CpG groups covering the targeted CpG island found in *Gpx1* comparing sedentary condition (SED, n = 7–8 mice) and exercise at time of sacrifice during their physically active time frame (EX (t = 2:00), n = 3). For reference, see daily running activity on Fig 1. Data are mean ± SEM, n = 3–8 mice. *: p<0.05 *versus* SED (Unpaired t-test).

To assess whether exercise could induce rapid changes in DNA methylation immediately after an exercise bout, we sacrificed WT mice during the last hour of their daily running activity (2:00 AM; Fig 1B), rather than during the day when they are inactive (Fig 1B). Interestingly, when methylation levels were measured in active WT mice (at t = 2:00), we observed that CpG regions 2 and 4 were demethylated during exercise, CpG region 1 tended to be demethylated, while region 3 remained unaffected (Fig 5). By comparison, methylation of none of these regions was affected in trained WT mice sacrificed during their inactive time (Fig 4B). This demonstrates the highly dynamic nature of epigenetic regulation.

### *In vitro* methylation decreases gene expression

**Fig 6:**
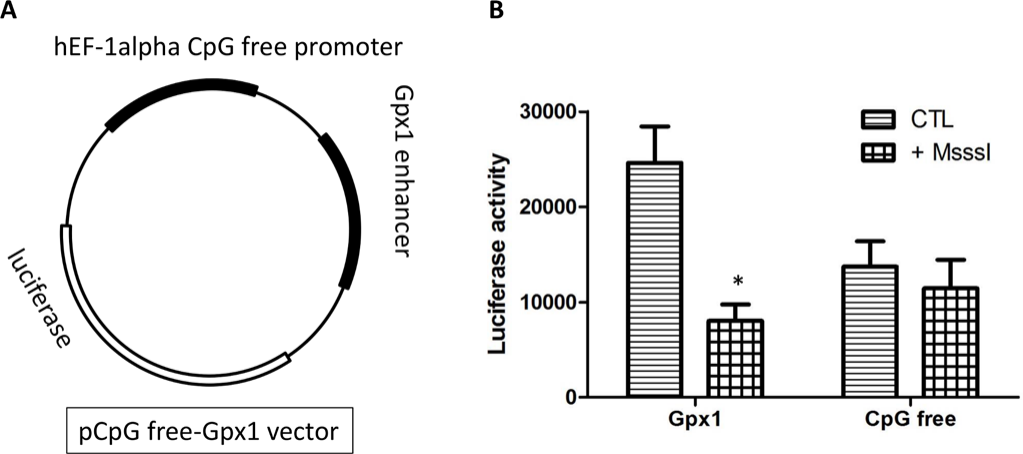
DNA methylation of *Gpx1* decreases gene expression. *In vitro* methylation of *Gpx1* target region inhibited transcriptional activity, as measured by a luciferase reporter assay. (A) Schematic representation of the plasmid construction containing the *Gpx1* CpG island region. (B) Luciferase activity ratio of methylated (M.SssI treated) to unmethylated control (CTL) plasmids containing a CpG-free promoter or the *Gpx1* CpG island region. The assay was repeated 4 times and data are mean ± SEM. *: p<0.05 *versus* CTL (Unpaired t-test).

To investigate the direct effect of DNA methylation on gene expression, the CpG-rich region of Gpx1, along with a CpG free promoter, was subcloned into the CpG-free-basic lucia vector. This new construction, CpG free-Gpx1 (Fig 6A), was methylated and transiently transfected in HEK293 cells to measure luciferase activity. The addition of the CpG-rich region significantly increases promoter activity (Fig 6B). Previously methylated vectors show no increase in promoter activity, similarly to empty vectors (Fig 6B). Therefore, methylation of this region is expected to inhibit *Gpx1* expression.

### No sign of oxidative stress in the skeletal muscle of dyslipidemic mice

**Fig 7:**
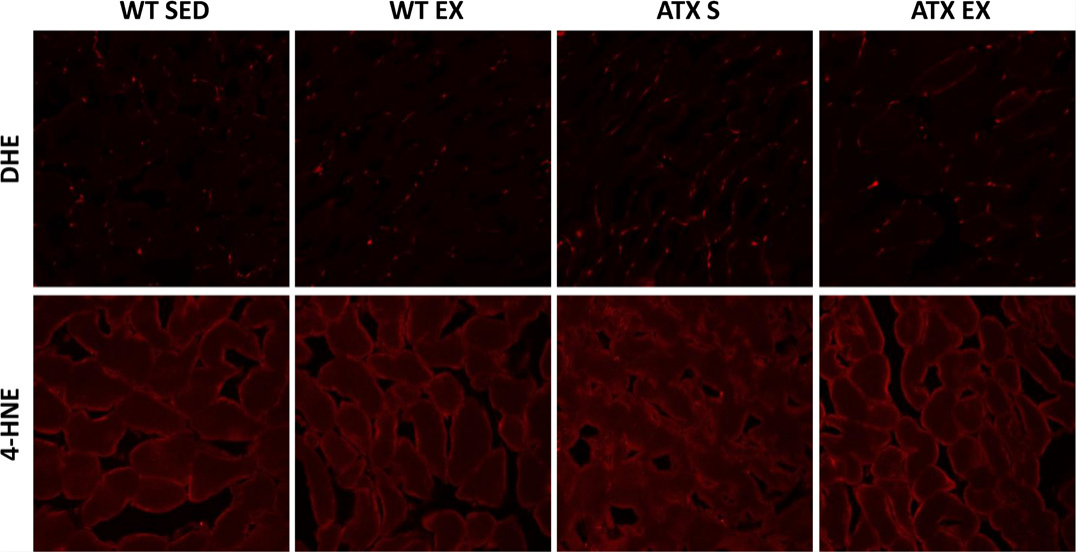
Oxidative stress estimation. Oxidative stress was measured by the fluorescent dye DHE (top panels) and the expression of 4-HNE (bottom panels) following staining of skeletal muscle cross-sections cut from (left to right) WT SED, WT EX, ATX SED and ATX EX mice.

Assessment of superoxide production by DHE staining and quantification of lipid peroxidation by 4-HNE immunostaining, in the skeletal muscle reveals similar oxidative stress between ATX and WT mice across all conditions (Fig 7), suggesting an efficient antioxidant compensation at this age.

## Discussion

Our findings indicate that the expression of *Gpx1* in the skeletal muscle is sensitive to the unstable *redox* environment associated with severe dyslipidemia and exercise. We also provide evidence that its expression is associated with epigenetic regulation, namely DNA methylation of a novel locus located in a coding region of the gene.

To generate energy, mitochondria exert oxidative phosphorylation. This process needs to be tightly regulated since electrons leakage from the electron transport chain reacts to oxygen generating superoxide. Uncoupling proteins (UCP) were first named as such for their ability to carry protons and steers the respiratory chain away from ATP synthesis in favour of thermogenesis [39]. UCP1 is only expressed in the brown adipose tissue whereas UCP2 and UCP3 are also found in the skeletal muscle where they play a role in fatty acid metabolism [40]. Regarding their role in ROS production and oxidative stress regulation, knock-down experiments in mice have shown increases in ROS production linked to deficiency in UCP2 [41] and UCP3 [42]. Our study reveals that expression of *UCP2*, but not *UCP3*, is lower in the skeletal muscle of ATX mice, suggesting that ROS production is favoured under dyslipidemia and that oxidative stress is more prone to happen.

Dysregulation of NOX activity is another well-known source of ROS in association with systemic pathological conditions including atherosclerosis [43] as well as diseases specific to the skeletal muscle [44, 45]. Importantly, NOX are expressed in both arteries and skeletal muscle precursor cells [46]. NOX2 and NOX4 regulate basal ROS production and although they are generally associated with inflammation, there is a controversy regarding their role in cell signalling, suggesting that there is an influence of the cell type and context [5]. In our study, we observed that exercise induces *Nox2* expression in the skeletal muscle of both WT and ATX mice without changes in *Nox4*. Although we did not assess whether the overexpression of *Nox2* is deleterious or beneficial to the skeletal muscle, this up-regulation of *Nox2* by exercise is somewhat surprising given the reports linking NOX2 to atherosclerotic lesions progression [47]. The role of NOX2 in the skeletal muscle is unclear, but recent reports have shown that the skeletal muscle contractile response triggers ROS production through a NOX2-dependent pathway [48] in addition to evidence for NOX2-mediated superoxide production in the aorta of rats following acute exercise [49]. Therefore, the contribution of ROS-generating enzyme to the level of oxidative stress in the presence of chronic severe dyslipidemia is only suggested by a change in their expression since non-specific DHE staining did not detect changes in ROS levels and 4-HNE immuno-histological staining did not show changes in lipid peroxidation. Antioxidant defense mechanisms are therefore expected to be up-regulated in these young dyslipidemic mice.

Expression of antioxidant enzymes is very dynamic, and changes in both expression and activity have been linked to atherosclerosis [50–52] as well as exercise [53, 54] and are determinant for preserving cellular functions [55–57]. In the present study, we found that expression of each antioxidant enzyme responded differently to the environmental context. *Trx1*, *Sod2* and *Prdx3* are all up-regulated in the skeletal muscle of sedentary ATX mice, and this was prevented by exercise, except for *Prdx3*. The increase in these antioxidant enzymes suggests a pro-oxidative state requiring an antioxidant response from the organ to prevent oxidative damage. These findings are consistent with other reports showing an increase in antioxidant enzymes expression during the onset and development of oxidative stress-related diseases [58, 59]. Exercise seems to lessen the amplitude of the antioxidant response by preventing the overexpression of *Trx1* and *Sod2* only. The mechanistic origin of this regulation is however unknown. Nonetheless, because exercise normalized their level of expression, this is likely secondary to a reduction in oxidative stress. If these two enzymes were responsible for the antioxidant effects of exercise, we would anticipate that their levels had increased above sedentary levels measured in WT mice.

In contrast to *Trx1* and *Sod2*, we observed that *Gpx1* varies in an opposite manner: not only its expression is decreased in ATX mice, it is also increased by exercise above basal levels in both WT and ATX mice. A study has previously shown that low activity of Gpx1 is an independent risk factor of cardiovascular events [60]. The increase in *Gpx1* with exercise is also coherent with the literature [61, 62] and concords with the known antioxidant properties of chronic exercise [16, 17]. Therefore, our data, in support of the current knowledge, suggest that overexpression of *Gpx1* is one of the mechanisms responsible for the antioxidant protection by exercise.

Our next aim was to test the hypothesis that the chronic changes in expression of the antioxidant enzymes were driven by DNA methylation. In our study, DNA methylation was undetectable for *Trx1* and *Prdx3* and did not vary between conditions for *Sod2*. *Gpx1*, however, exhibited significant levels of methylation that were increased in ATX mice. Recent studies have shown that this gene could be regulated by DNA methylation of regions near the promoter in cancer cells, where a hypermethylation of *Gpx1* and the consequent silencing of the gene disturb the *redox* environment, favouring progression of the disease [63–65]. In line with these studies, we observed that hypermethylation of this novel CpG-rich region was associated with a reduced *Gpx1* expression, in the context of dyslipidemia.

The *in vitro* methylation assay allowed us to confirm that methylation of this specific locus causes an inhibition of gene expression. By itself, the CpG-rich region increases gene activity and when methylated, we observe transcriptional activity similar to that of a *Gpx1*-free sequence. This suggests that methylation of the CpG island element blocks the binding and subsequent activity of a transcription co-factor. An interesting finding is that although *Gpx1* expression responds to both dyslipidemia and exercise, only changes in methylation are detected with dyslipidemia and not exercise, at least not in the long-term. This finding could contradict various studies that show changes of DNA methylation in response to exercise for various genes in the skeletal muscle [66–68]. Alternatively, changes in *Gpx1* expression in response to physical training may be modulated by mechanisms other than epigenetics. Albeit this cannot be dismissed, a previous study in human skeletal muscle showed that acute exercise induces transient changes in the methylation of genes with changes in expression only observed later, once methylation changes reverted to pre-exercise levels [69]. This mismatch in the timing of both events (changes in methylation and expression) could have occurred in our study. Exercise could induce temporary epigenetic changes that would have disappeared as little as minutes after the muscle work. In our first series of experiments, sacrifice of the animal was performed at 10 AM, several hours after the last running session. Hence, to assess the methylation profile in mice during physical activity, we sacrificed mice at 2 AM (t = 2:00) when they are still running (Fig 1B); in these conditions we observed methylation changes that were not detected in our previous experiments, demonstrating the temporary changes in methylation induced by physical exercise. Altogether, these data suggest that epigenetic mechanisms could be responsible for the changes in expression in response to exercise that we observed for *Gpx1* in the long-term. Further investigation is required to understand the link between the initial transient changes in DNA methylation that occurs during exercise bouts and lasting changes in gene expression observed in the long-term.

Exacerbation of oxidative stress in the skeletal muscle cells in the context of dyslipidemia is caused by a disturbance in the production and elimination of mitochondria-derived free radicals [13]. This, in turn, impairs the organ, and the excess of ROS can diffuse to the femoral vascular bed causing vascular endothelial dysfunction, consequently impeding blood flow regulation to the muscle leading to sarcopenia [13]. This vicious circle has previously been observed in hypertensive rats [70]. In our experimental conditions, there is no evidence of oxidative stress-dependent damage in the skeletal muscle of our young, middle-age ATX mice, suggesting that oxidative stress is well compensated in skeletal muscle, supporting the adaptive responses of antioxidant gene expression reported above. This is confirmed by the demonstration that in these mice, skeletal muscle function was not altered as evidenced by their conserved running capacity.

### Limitations of the study

The nature of voluntary exercise makes it difficult to detect the minute changes in the methylation landscape in the long-term, if any. First, the impact of voluntary running used in the present study could be considered as mild when compared to other types of experiments in the field of exercise training: “forced” approaches such as treadmill or swim tests might be able to induce greater and more stable epigenetic changes. However, one could argue that the stress occurring during these forced bouts of exercise could have a significant impact on the observation. We chose to rule out the stress factor with our voluntary approach, knowing the significant impact of stress on epigenetics [71, 72]. Secondly, a possible explanation for the discrepancies between dyslipidemia and exercise on epigenetics is that the former is a long-term and continuous stimulation, as opposed to a short-term and intermittent stimulation for the latter. In other words, mice are constantly exposed to dyslipidemia from *intra utero* but they only practice intermittent voluntary bouts of exercise, starting at 3-month of age. Studies in the field of developmental plasticity have shown that *in utero* and early life exposure to environmental factors induces phenotypic adaptations that are reflected through adulthood and that these changes are deeply rooted in the epigenome [73, 74]. Protein expressions of Gpx1 reveal yet another level of complexity and do not fully reflect the changes in mRNA levels in ATX mice. This is likely associated with differential post-translational regulation in dyslipidemia beyond the scope of the present study. Finally, whether ROS are responsible for the epigenetic changes observed both with dyslipidemia and during exercise was not directly tested. The use of an antioxidant may help, although they most likely may directly impact on the epigenome.

## Conclusions

The overexpression of some pro- and anti-oxidant enzymes by severe dyslipidemia suggests a pro-oxidative state in the skeletal muscle where the chronic rise in ROS could damage skeletal muscle cells, to latter contribute to the damage of the neighbouring vascular cells. We show that one of the mechanisms by which the antioxidant enzyme Gpx1 is modulated is by DNA methylation; its lower expression is associated with the higher level of methylation of a novel CpG island region in ATX mice. We also reinforced the cause-effect relationship between DNA methylation of this loci and its mRNA expression. Our data suggests, therefore, that DNA methylation is a possible mechanism for regulating gene expression in dyslipidemia as well as following chronic voluntary exercise training. Whether ROS are directly involved remains debatable.

## Supporting Information

S1 Fig Gpx1 protein expression. (PDF)

S2 Fig CpG methylation analysis of *Prdx3*, *Sod2* and *Trx1*. (PDF)

S1 Table Primer sequences used for qPCR analysis. (PDF)

S2 Table Primer sequences used for DNA methylation analysis. (PDF)
