# Supplementary material for: Epigenetic Regulatory Effect of Exercise on Glutathione Peroxidase 1 Expression in the Skeletal Muscle of Severely Dyslipidemic Mice
Source: PLoS One. 2016 Mar 24;11(3):e0151526. doi: 10.1371/journal.pone.0151526 (PMC4806847; doi:10.1371/journal.pone.0151526)
Supplement: S1 Fig — (PDF) [file pone.0151526.s001.pdf]

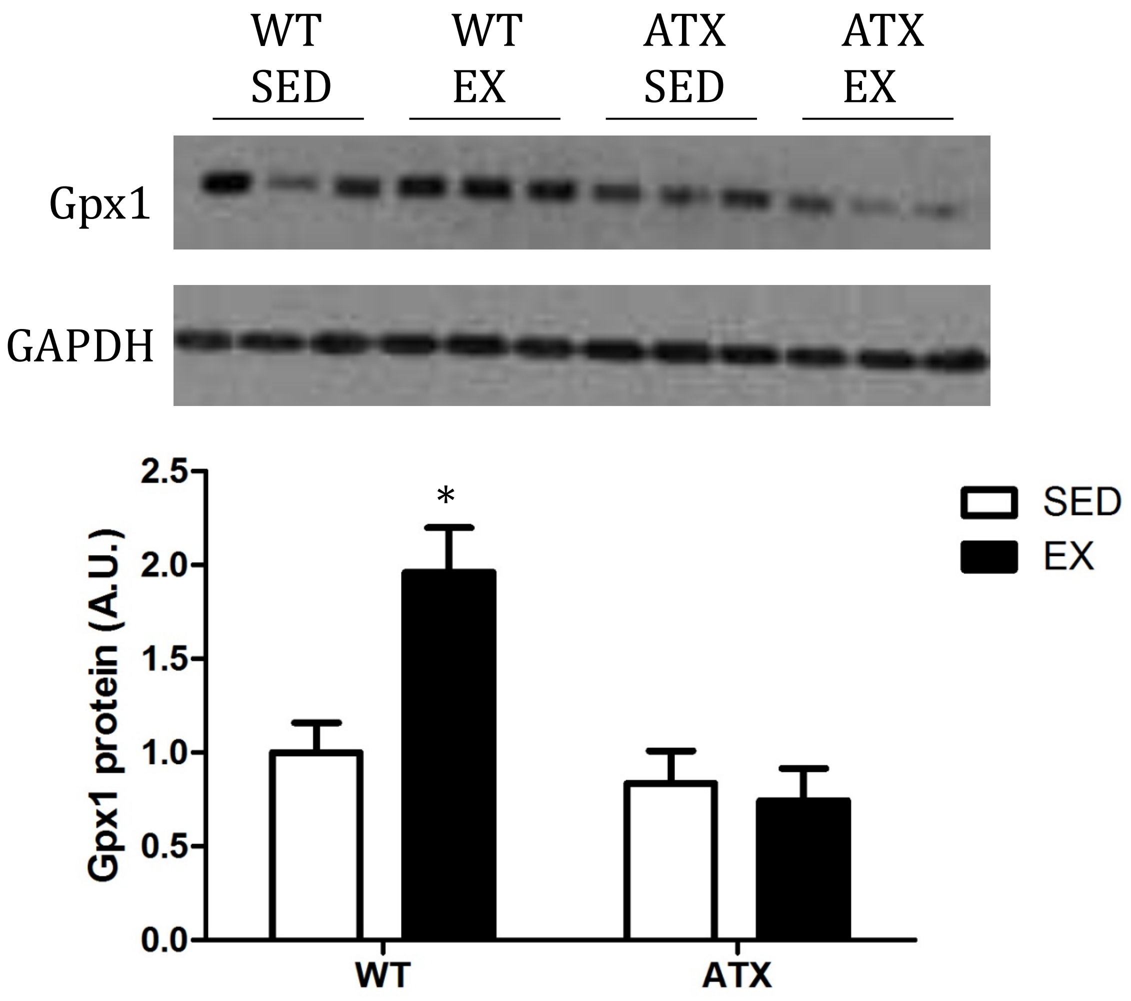

S1 Fig. Gpx1 protein expression. Protein were extracted from skeletal muscle of wild-type (WT) and dyslipidemic (ATX) mice in the sedentary (SED) and exercise (EX) groups. Data are mean ± SEM, n=6 mice per group. *: $p<0.05$ *vs.* WT-SED (Two-way ANOVA).
