# Supplementary material for: Epigenetic Regulatory Effect of Exercise on Glutathione Peroxidase 1 Expression in the Skeletal Muscle of Severely Dyslipidemic Mice
Source: PLoS One. 2016 Mar 24;11(3):e0151526. doi: 10.1371/journal.pone.0151526 (PMC4806847; doi:10.1371/journal.pone.0151526)
Supplement: S2 Fig — (PDF) [file pone.0151526.s002.pdf]

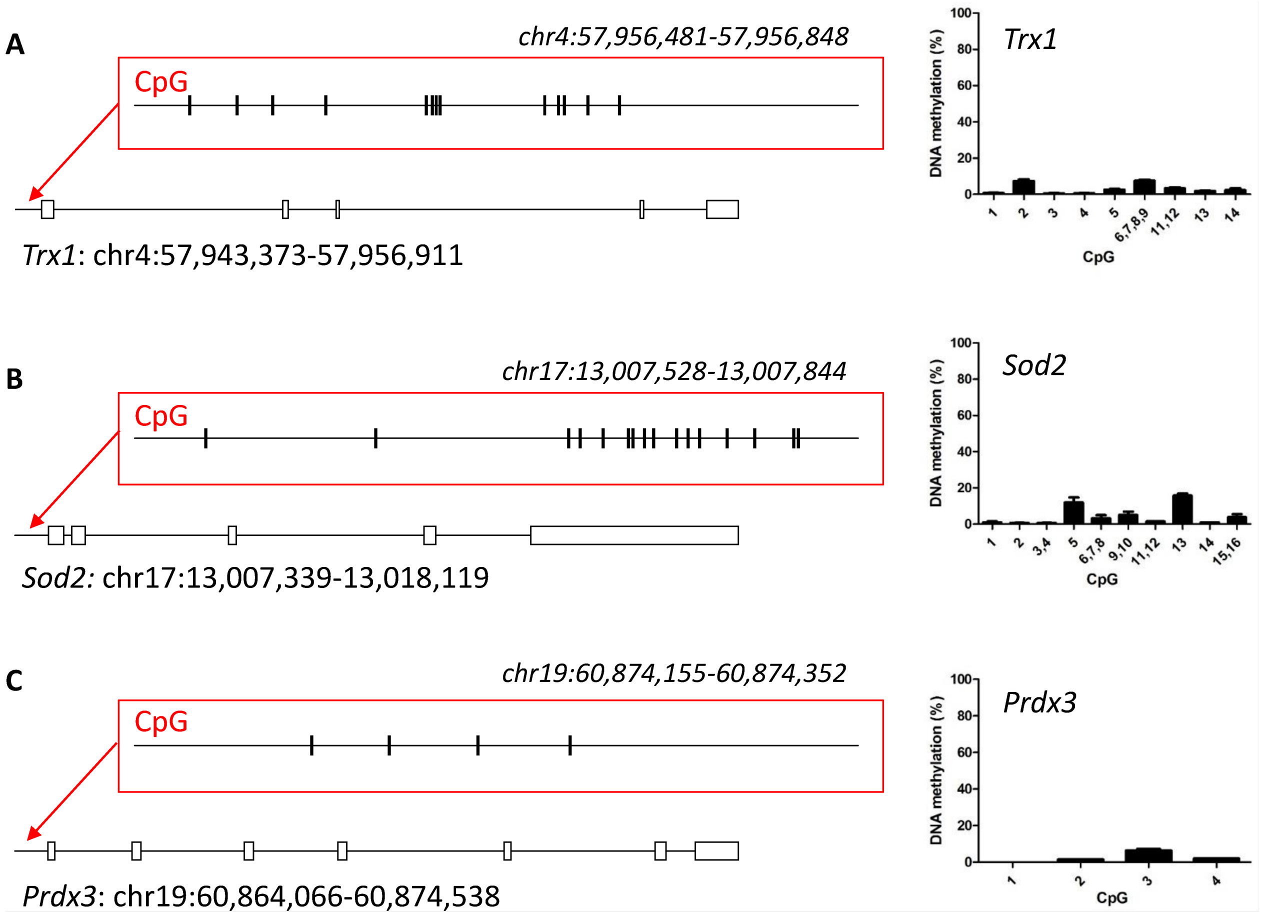

S2 Fig. CpG methylation analysis of *Prdx3*, *Sod2* and *Trx1*. *Prdx3*: peroxiredoxin 3; *Sod2*: superoxide dismutase 2, mitochondrial; *Trx1*: thioredoxin 1.
