# Supplementary material for: Epigenetic Regulatory Effect of Exercise on Glutathione Peroxidase 1 Expression in the Skeletal Muscle of Severely Dyslipidemic Mice
Source: PLoS One. 2016 Mar 24;11(3):e0151526. doi: 10.1371/journal.pone.0151526 (PMC4806847; doi:10.1371/journal.pone.0151526)
Supplement: S1 Table — (PDF) [file pone.0151526.s003.pdf]

S1 Table. Primer sequences used for qPCR analysis. UCP2/3: uncoupling protein (mitochondrial proton carrier) 2/3; NOX2/4: NADPH oxidase 2/3; Trx1: thioredoxin 1; SOD2: superoxide dismutase 2, mitochondrial; Prdx3: peroxiredoxin 3; Gpx1: glutathione peroxidase 1.

**Gene**	**Primer sequence**
UCP2	(F) CTCGTCTTGCCGATTGAAGGT<br>(R) TCTGCAATGCAGGCAGCTGTC
UCP3	(F) GAGAAGTTGCTGGAGTCTCAC<br>(R) GAGCCACCATCTTCAGCATAC
NOX2	(F) AGTGCCCAGTACCAAAGTTC<br>(R) GTCCCACCTCCATCTTGAATC
NOX4	(F) CCTTTGTGCCTTTATTGTGCG<br>(R) GAATCGTTCTGTCCAGTCTCC
Trx1	(F) AATGGTGAAGCTGATCGAGAC<br>(R) TTTCCTTGTTAGCACCGGAG
SOD2	(F) GGCCAAGGGAGATGTTACAA<br>(R) GCTTGATAGCCTCCAGCAAC
Prdx3	(F) TCGGTATCTCCGCCTATCGT<br>(R) GAGGAACTGGTGCTAAAGGCT
GPx1	(F) CACCGAGATGAACGATCTG<br>(R) CAGGTCGGACGTACTTGAG
