# Supplementary material for: Epigenetic Regulatory Effect of Exercise on Glutathione Peroxidase 1 Expression in the Skeletal Muscle of Severely Dyslipidemic Mice
Source: PLoS One. 2016 Mar 24;11(3):e0151526. doi: 10.1371/journal.pone.0151526 (PMC4806847; doi:10.1371/journal.pone.0151526)
Supplement: S2 Table — (PDF) [file pone.0151526.s004.pdf]

S2 Table. Primer sequences used for DNA methylation analysis. Gpx1: glutathione peroxidase 1; Prdx3: peroxiredoxin 3; Sod2: superoxide dismutase 2, mitochondrial; Trx1: thioredoxin 1.

Gene	EPITYPER Primer sequence
Gpx1	(F)aggaagagagGGGATTTTGAGATTTAGAGTTGGTT<br>(R)cagtaatacgactcactatagggagaaggctATTCTCAATAAACAACACCTTACCC
Prdx3	(F)aggaagagagTTAGGTTTGGGTGTTTTAGTGAAGA<br>(R)cagtaatacgactcactatagggagaaggctCCCTTAAATCTACTACCCACTAACTCC
Sod2	(F)aggaagagagATGTTAGGTTAGGTTTTAGGGAAGG<br>(R)cagtaatacgactcactatagggagaaggctTCCCCTATACCAAATTAATAAAAACC
Trx1	(F)aggaagagagTTAGTGTAGTTTGGGGATTGTTTTA<br>(R)cagtaatacgactcactatagggagaaggctCCCTAATCTAAAAATACACCTCTACC
